# Supplementary material for: Hybrid Models and Biological Model Reduction with PyDSTool
Source: PLoS Comput Biol. 2012 Aug 9;8(8):e1002628. doi: 10.1371/journal.pcbi.1002628 (PMC3415397; doi:10.1371/journal.pcbi.1002628)
Supplement: Text S4 — Complete source code for the PyDSTool package (version 0.88.120504). Includes API documentation and help files linking to web pages. This file is identical to the current public release on Sourceforge.net. (ZIP) [file pcbi.1002628.s004.zip › PyDSTool/html/toc-PyDSTool.Toolbox.ParamEst-module.html]

xml version="1.0" encoding="ascii"?


ParamEst


# Module ParamEst

---

## Classes

BoundMin  
 ConstraintFail  
 Converged  
 L2\_feature  
 L2\_feature\_1D  
 LMpest  
 ParamEst  
 residual\_fn\_context  
 residual\_fn\_context\_1D  

## Functions

\_makeUnique

\_present\_and\_sensitive

compare\_data\_from\_events  
 ctn\_residual\_info  
 do\_2Ddirn  
 do\_2Dstep  
 filter\_feats  
 filter\_iface  
 filter\_pars  
 get\_extrema  
 get\_extrema\_from\_events  
 get\_slope\_info  
 grad\_from\_psens  
 make\_opt  
 norm\_D\_sum  
 organize\_feature\_sens  

pp

restrict\_opt  
 select\_pars\_for\_features  
 sweep1D  

## Variables

HAVE\_PSYCO

Tester  

\_ctn\_functions

\_deprecated\_functions

\_errors

\_generic\_opt

\_pest\_classes

\_seq\_types

\_utils

power

rerr

res\_fn\_lookup

rout

sign

solver\_lookup

test  

---

[hide private]
